# Ultrasound‐Assisted Extraction Using a 3D‐Printed Device Functionalized With SPE Sorbent for UHPLC‐DAD Analysis of Pesticide Residues in Honey

**DOI:** 10.1111/1750-3841.70788

**Published:** 2025-12-28

**Authors:** Daniela Lupu, Gabriel Hancu, Laura Ferrer

**Affiliations:** ^1^ Environmental Analytical Chemistry Group University of the Balearic Islands Palma Spain; ^2^ Department of Pharmaceutical and Therapeutic Chemistry, Faculty of Pharmacy, “George Emil Palade” University of Medicine, Pharmacy Science and Technology of Târgu Mures Târgu Mures Romania

**Keywords:** 3D‐printed device, honey, pesticides, SPE, UHPLC, UAE

## Abstract

The presence of pesticide residues in honey represents a significant concern for food safety and public health, underscoring the need for advanced analytical techniques capable of ensuring accurate detection and effective food control. In this study, a stereolithographically 3D‐printed device coated with the solid‐phase extraction (SPE) sorbent Oasis MCX was developed for the ultrasound‐assisted extraction (UAE) of pesticide residues in honey, followed by ultra‐high performance liquid chromatography with diode array detection (UHPLC‐DAD) analysis. Variables that affect the performance of the 3D‐printed extraction (i.e., design and fabrication of 3D‐printed device, SPE resin, elution solution, pH, and ultrasonic conditions) and the chromatographic analysis were systematically evaluated. The optimized methodology allowed the simultaneous determination of five pesticides: azoxystrobin (AZO), carbaryl (CAR), pirimicarb (PIR), thiacloprid (THC), and thiamethoxam (THI). Chromatographic separation was completed in less than 15 min using a KinetexXB‐C18 column (2.6 µm, 100 Å, 100 × 3 mm), with a mobile phase consisting of methanol and 0.1% formic acid (pH 3.35). The proposed methodology showed satisfactory agreement compared with commercial QuEChERS dispersive SPE kits. The developed method demonstrated good linearity up to 100 mg L^−1^ (*R^2^* > 0.99), low detection limits (0.002–0.5 mg kg^−1^), good precision (< 5.1 % RSD), and satisfactory recoveries (90–109 %). Its successful application to real honey samples demonstrates the method's potential for reliable multi‐residue pesticide analysis in complex food matrices. The sample preparation stage was evaluated with the AGREEprep tool, yielding a greenness score of 0.43, which indicates a moderately green performance.

## 1 Introduction

The presence of pesticide residues in agricultural commodities has become a critical global concern due to the significant risks they pose to both human health and environmental sustainability (Kaur et al. 2024; Lebelo et al. 2021; Andreotti et al. 2018). In 2021 alone, approximately 355,000 tons of pesticides were sold across the European Union (EU), with the highest volumes reported in Germany, France, Spain, and Italy, countries that also rank among the EU's leading agricultural producers (European Environment Agency 2023). As persistent organic pollutants, pesticides are released into the environment via soil, water, and air, ultimately accumulating throughout the food chain (Alburaki et al. 2015; Chiesa et al. 2016; Li et al. 2017). Even when pesticide levels remain below regulatory thresholds, chronic exposure may still pose significant health risks (Sharma et al. 2023). Organophosphates and carbamates, for instance, are neurotoxic compounds linked to neurodegenerative disorders like Parkinson's disease. (Damalas and Eleftherohorinos 2011). Strobilurin fungicides have been associated with nephrotoxicity and an increased incidence of kidney cancer in exposed populations (Feng et al. 2020), with synergistic toxic effects observed when combined with neonicotinoids (Wei et al. 2021). Carbamates such as PIR and CAR raise additional concerns due to their cytotoxic and genotoxic properties, as well as their effects on neuronal development and male fertility, including reduced semen quality (Saquib et al. 2021). Similarly, neonicotinoids like THI are under renewed scrutiny following reports of acute kidney injury and systemic toxicity in humans (Ramanathan et al. 2020; Zhang et al. 2024).

Honey, widely considered a safe and natural product, serves as a key matrix for monitoring pesticide residues due to its susceptibility to contamination from environmental sources such as agricultural runoff and spray drift (Bedi et al. 2015; Carrasco Cabrera et al. 2023; Hassanin et al. 2018). These contaminants have been frequently detected not only in honey but also in related bee‐derived products, including pollen, beeswax, and beebread (El Agrebi et al. 2019; Al Naggar et al. 2015; Panseri et al. 2014; Ravoet et al. 2015). Their presence raises serious concerns regarding both food safety and pollinator health. Exposure to pesticide residues can compromise bees’ immune function and increase mortality rates, thereby threatening pollinator populations essential to global food production (Samson‐Robert et al. 2014).

Pesticide contamination in honey can occur during foraging and persist throughout processing, compromising product quality and posing economic risks (Ashraf et al. 2023; Irungu et al. 2016). Agriculture remains the primary source of contamination, as residues are transferred from treated crops to nectar and pollen (Cappellari et al. 2024; Kasiotis et al. 2023; Kumar et al. 2018). However, non‐agricultural applications, environmental runoff, and certain beekeeping practices also contribute to contamination (Fuente‐Ballesteros et al. 2023; Kavanagh et al. 2021). Both bees and honey are therefore recognized as effective bioindicators for monitoring environmental pesticide exposure (Hung and Yiin 2023; Przyby and Wilczyńska 2001). The accumulation of pesticide residues within beehives has been strongly linked to increased bee mortality and the occurrence of Colony Collapse Disorder (Calatayud‐Vernich et al. 2019; Leska et al. 2021).

Although regulatory frameworks differ among countries, maximum residue limits (MRLs) have been established to minimize potential risks to human health (FAO 2024). The most frequently detected classes of pesticides in honey include neonicotinoids, carbamates, pyrethroids, and strobilurins (Harwood and Dolezal 2020). Frequently reported individual compounds are THC, THI, CAR, PIR, and AZO, each characterized by distinct chemical structures and physicochemical properties (Table S1, Supplementary material), (Baša Česnik et al. 2019; Jiang et al. 2018; Lozano et al. 2019). Recent advancements have focused on the miniaturization of sample preparation by optimizing key parameters involved in the extraction process of pesticides from honey products. These parameters include extraction time, sample volume, solvent composition and volume, pH, and salt additions. Among the available methods, quick, easy, cheap, effective, rugged, and safe (QuEChERS) has become the most widely used approach in recent decades. QuEChERS extraction is widely applied in food safety analysis, and it has been adapted for honey to solve some drawbacks such as incomplete matrix cleanup (Niell et al. 2015). Modified QUEChERS has been successfully applied to the determination of pesticide residues in both honeybees and honey (Makni et al. 2023; Musarurwa et al. 2019; Barganska et al. 2014).

In addition, other miniaturized extraction techniques, such as dispersive liquid–liquid microextraction (DLLME) and solid‐phase microextraction (SPME), have also gained increasing attention. These methods allow for the analysis of a broad spectrum of compounds, both polar and non‐polar, while offering advantages such as reduced analysis time, fewer procedural steps, and lower reagent consumption. As a result, these approaches consistently yield high recovery rates. Subsequent separation, identification, and quantification of analytes are typically performed using chromatographic techniques (Kerkich et al. 2024).

However, the complex composition of honey, characterized by high levels of sugars, proteins, and polyphenols, presents significant challenges for both extraction and quantification (Souza Tette et al. 2016). Matrix effects, high viscosity, and the potential for analyte degradation necessitate carefully optimized sample preparation protocols (Cortese et al. 2020).

In this sense, ultrasound‐assisted extraction (UAE) has gained considerable attention for its ability to enhance extraction efficiency, particularly in viscous or heterogeneous matrices. By exploiting acoustic cavitation effects, UAE promotes solvent penetration, accelerates analyte release, and significantly reduces extraction time. Consequently, it represents a greener and more efficient alternative to conventional extraction techniques (Du et al. 2022; Shen et al. 2023).

Recently, 3D‐printing has found growing application in analytical chemistry through the development of customized SPE‐coated devices. These tools have been successfully employed for monitoring various pollutants, including antibiotics and nonsteroidal anti‐inflammatory drugs, and offer key advantages such as controllable porosity, design flexibility, and reusability (Gross et al. 2017). As such, they hold significant potential for trace‐level analysis in both environmental and food matrices (Barzallo et al. 2023; Vargas‐Muñoz et al. 2025). The incorporation of 3D‐printed structures as supports for SPE offers clear analytical and practical advantages over conventional sorbent formats. Additive manufacturing enables precise control of geometry and surface architecture, yielding reproducible microstructures that promote efficient sorbent coating and uniform analyte–sorbent interactions. These features improve mass transfer, reduce diffusion limitations, and enhance extraction efficiency. Additionally, 3D printing facilitates miniaturization, customization, and reduced sorbent and solvent consumption, improving reproducibility, operational simplicity, and sustainability in line with current trends in green and advanced sample preparation (Monteiro et al. 2023).

In this study, stereolithography (SLA) 3D printing was used to fabricate a reusable SPE support with a precisely engineered micro structured surface. Its regularly patterned quadrilateral features enable uniform resin deposition and maximize active surface area, enhancing mass transfer, coating homogeneity, and extraction efficiency compared with flat supports.

To the best of our knowledge, this is the first study to integrate UAE with a 3D‐printed device coated with an SPE resin for the extraction of pesticide residues in honey followed by UHPLC‐DAD analysis. This innovative approach enables the selective and simultaneous extraction, separation, and quantification of five target pesticides, i.e., AZO, CAR, PIR, THI, and THC. The method was designed to enhance analytical accuracy and address the challenges associated with the complex honey matrix. The AGREEprep tool was employed to evaluate the environmental impact of the proposed sample preparation procedure (Wojnowski et al. 2022).

## 2 Materials and Methods

### 2.1 Reagents and Materials

All reagents used were of analytical grade. Acetonitrile (ACN), methanol (MeOH) (99.8%), polyvinylidene fluoride (PVDF), dimethylformamide (DMF, 99.5%), formic acid, 2‐propanol, and sodium chloride (NaCl) were purchased from Scharlab (Barcelona, Spain). Analytical standards of pesticides (AZO, CAR, PIR, THI, and THC) were obtained from Sigma‐Aldrich (Darmstadt, Germany). Individual stock solutions of each pesticide were prepared at a concentration of 2500 mg L^−1^ by dissolving 10 mg of each compound in 4 mL of an appropriate solvent, selected according to the solubility characteristics of each pesticide. Specifically, AZO was dissolved in ACN, PIR, and THC in MeOH, and CAR and THI in a 1:1 (v/v) ACN–water mixture. A mixed pesticide standard solution was subsequently prepared at a final concentration of 100 mg L^−1^ for each pesticide by combining appropriate volumes of the individual stock solutions.

QuEChERS dispersive SPE (dSPE) kits were acquired from Phenomenex (Madrid, Spain). Ultrapure water (18.2 MΩ·cm) was produced using a Milli‐Q water purification system (Millipore Ibérica, Madrid, Spain). The 3D‐printed device was fabricated using Clear V4 photoactive resin (FLGPCL04) (Formlabs Inc., USA). Oasis MCX cartridges were obtained from Waters (Milford, MA, USA).

### 2.2 Software and Instrumentation

The computer‐aided design (CAD) of the devices was carried out using Rhinoceros 6 software (McNeel and Associates). Devices were printed with a FormLabs 3 + SLA printer (Somerville, MA, USA). Design preparation for 3D‐printing was performed with PreForm software, version 3.25. Post‐printing, the devices were cured under UV light using a CL‐1000 ultraviolet crosslinker equipped with a 365 nm lamp (Champaign, IL, USA). An ultrasonic bath sonicator (Labbox, ULTR 6L5‐001) was employed for sample preparation. Quantification was conducted using a Vanquish Core UHPLC system (Thermo Fisher Scientific, MA, USA) coupled with a diode array detector (DAD) operating across the 190–800 nm wavelength range.

### 2.3 Chromatographic Conditions

UHPLC‐DAD analysis was performed using a Vanquish core UHPLC system (Thermo Fisher Scientific) equipped with a quaternary pump and an auto sampler. Data acquisition and processing were carried out using Chromeleon 7.2 software (Thermo Scientific). Chromatographic separation was achieved in less than 15 min using a KinetexXB‐C18 column (2.6 µm, 100 × 3 mm, 100 Å), with a mobile phase consisting of MeOH and 0.1% formic acid (A) and 0.1% formic acid in water, pH 3.35 (B), operated in gradient mode, at a flow rate of 0.25 mL min^−1^. Measurements were registered at 228 nm. The UHPLC‐DAD method was systematically optimized using a design of experiments (DoE) approach to achieve efficient and robust chromatographic separation. A preliminary screening was based on a factorial design to evaluate key chromatographic parameters, including mobile phase composition, gradient profile, column temperature, and flow rate. Two solvent systems and three reverse‐phase columns were tested. A Box–Behnken design was applied to investigate the influence of gradient time (8.0, 11.5, 15.0 min) and column temperature (30, 35, 40°C) on separation quality. The primary response variable was the minimum resolution (*Rs_min_*) between adjacent peaks, while secondary responses included the average retention time and theoretical plate number. A total of thirteen experimental runs were performed to model the response surfaces and identify optimal chromatographic conditions.

### 2.4 Design, Fabrication and Coating of the 3D‐printed Device

**FIGURE 1:**
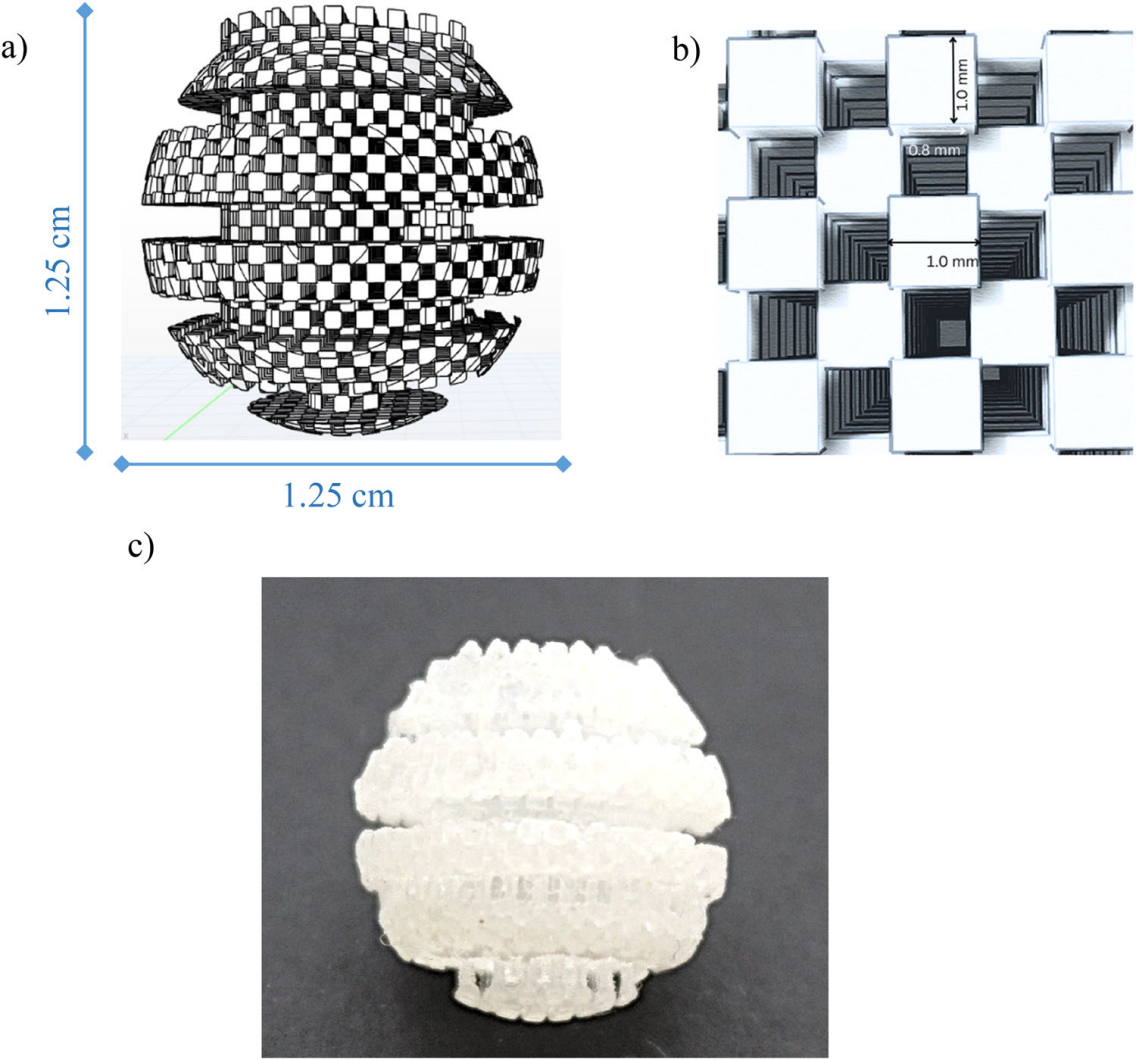
3D‐printed device (a) schematic representation illustrating the spatial configuration and surface topology designed to enhance sorbent–sample interactions; (b) detailed view of the structured extraction head with small cubic cavities (1 × 1 mm), arranged in an alternating binary pattern; and (c) real image.

The design of the 3D‐printed device was inspired by the structure of a traditional honey spoon, intended to enhance the interaction between the sample and the sorbent‐coated surface (Figure 1). The 3D‐printed device features a spherical extraction head composed of layered horizontal segments, topped with a handling tab for easy manipulation. The surface is uniformly patterned with a grid of cubic elements arranged to form regularly distributed cavities. Each cube measures 1 mm per side and is spaced 0.8 mm apart. This configuration was designed to maximize surface area, ensuring effective sorbent coating and enhanced mass transfer efficiency. Clear V4 resin was selected due to its proven suitability for fabricating microstructured components used in analytical applications (Barzallo et al. 2023). Additionally, its transparency allowed visual inspection, while its mechanical and chemical stability ensured compatibility with post‐processing and sorbent immobilization procedures. The printing process required approximately 204 min to fabricate 13 devices, using 251 layers with adaptive layer resolution and consuming a total of 66.6 mL of resin. Following printing, the devices were immersed in 2‐propanol for 2 min to remove residual unpolymerized monomers.

For sorbent immobilization, the “stick‐and‐cure” technique was employed using the Oasis MCX resin. The uncured 3D‐printed devices were placed in Falcon tubes containing the resin and manually shaken to facilitate sorbent adhesion. A cartridge containing 60 mg of MCX resin was sufficient to coat five 3D‐printed devices (12 mg resin per device). Finally, the coated 3D‐printed devices were exposed to UV light curing for 240 min to ensure stable fixation of the sorbent onto the 3D‐printed support (Barzallo et al. 2023).

### 2.5 SPE Resin and Extraction Variables Selection

Several SPE resins, including C18, Bakerbond, Oasis HLB, Oasis MCX, and Strata‐X, were evaluated for the extraction of the five target pesticides based on their recovery efficiencies. Each resin was immobilized onto the 3D‐printed using the “stick and cure” technique. Extraction performance was assessed under the conditions recommended by manufacturers, using spiked honey samples as the test matrix.

Once the SPE resin was selected, several variables influencing the extraction of pesticides from honey samples were investigated through univariate assays, i.e., sample pH, type of elution solution, and retention and elution times. Additionally, two techniques have been evaluated to improve the extraction efficiency, i.e., magnetic stirring and UAE.

### 2.6 Extraction Procedure

Honey samples (∼5 g) were dissolved in 3 mL of Milli‐Q water in a glass beaker to obtain a homogeneous solution. The pH was adjusted to 5 ± 0.5 when necessary; however, most honey samples naturally exhibited pH values within the range (4.8–5.1) and therefore did not require adjustment. Subsequently, 7.5 mL of ACN was added, followed by the addition of 1.0 g of NaCl to induce phase separation. The mixture was vortexed for 1 min, and a 6.5 mL aliquot of the upper ACN phase was transferred into a 50 mL Falcon tube for subsequent extraction. The coated 3D‐printed device was preconditioned by aspirating and expelling 1.5 mL of MeOH through it with the aid of a Pasteur pipette to activate the sorbent surface. This step was standardized by controlling the dispensed volume within ± 0.1 mL and repeating the procedure twice to ensure reproducibility of the preconditioning process. The coated 3D‐printed device was then inserted into the Falcon tube containing the honey extract. The mixture was vortexed for 1 min, followed by sonication for 20 min in an ultrasonic bath to enhance analyte adsorption onto the sorbent. After the retention step, the coated 3D‐printed device was rinsed with 1 mL of ultrapure water to remove residual matrix components. Subsequently, it was transferred to a clean vial containing 1 mL of methanol with 0.1% formic acid (MeOH + 0.1% formic acid) for elution of the adsorbed analytes. The vial was vortex‐mixed for 1 min and sonicated for 20 min to ensure complete desorption of the analytes from the sorbent surface. The resulting extract was filtered through a 0.22 µm nylon syringe filter, transferred into an amber vial, and stored at 4°C until UHPLC‐DAD analysis. A schematic representation of the sample preparation workflow is provided in Figure S1.

### 2.7 Analytical Performance of UAE‐3D‐Printed‐UHPLC‐DAD Method

Method validation was performed in accordance with the SANTE/11312/2021 Revision 2 guidelines (EUR 2026), with a focus on performance parameters relevant to pesticide residue analysis.

Initially, solvent‐based calibration curves were constructed using the mixed pesticide standard solutions and analyzed under the same UAE–3D‐printed coated device extraction and UHPLC‐DAD conditions as the matrix‐matched standards. These curves (Figure S2a) served as the reference for evaluating matrix effects.

Subsequently, matrix‐matched calibration curves (Figure S2b) were prepared by fortifying commercial honey samples (previously confirmed to be pesticide‐free using the QuEChERS method) with known concentrations of the mixed pesticide standard solution prior to extraction. The fortified samples were then subjected to the entire sample preparation procedure, including UAE with the 3D‐printed coated device, following the same protocol as for analytical samples. Thus, potential matrix effects were minimized to ensure accurate quantification of pesticide residues in honey. All analyses were performed using matrix‐matched calibration using 0.05, 3.12, 6.25, 12.5, 25, 50, 75, and 100 mg L^−1^ standards. Although all levels were analyzed, only the most representative concentrations were displayed in Figure S2b for clarity of calibration curve visualization.

Methodological calibration curves were also employed to assess the linear working range, linearity, and method limits of detection (MLODs) and quantification (MLOQs) using reagent blanks. Recovery was calculated as the ratio between found and added concentrations and is expressed as recovery ranges obtained from triplicate analyses of honey samples spiked at three concentration levels within the linearity range (10, 30, and 50 mg L^−1^), corresponding to low, medium, and high fortification levels, respectively. Precision was evaluated in terms of relative standard deviation (RSD, %) from replicate analysis of the same sample using different 3D‐printed coated devices, considering intra‐day (*n* = 3) and inter‐day (*n* = 9) measurements to assess method reproducibility over time. The enrichment factor (EF) was calculated as the ratio between analyte concentration in the eluate and in the original sample, while preconcentration factor (PF) was calculated as the ratio between sample volume and the eluate volume. Matrix effects were quantified by comparing the slopes of calibration curves prepared in pure solvent and in blank honey extract. Selectivity was verified by analyzing blank honey samples and their corresponding spiked matrices.

Finally, the reusability of the 3D‐printed coated device was assessed by studying the extraction efficiency obtained in two successive extraction cycles performed with the same 3D‐printed device. Scanning electron microscopy (SEM) images were taken for the visual evaluation of the 3D‐printed devices.

### 2.8 QuEChERS UHPLC‐DAD Method

In this study, a modified QuEChERS protocol based on EN 15662 (Phenomenex 2018) was employed to evaluate and compare the performance of the 3D‐printed coated device for UAE‐based extracting pesticide residues from honey. Initially, 5 g of honey were dissolved in 7.5 mL of ultrapure water to obtain a homogeneous solution, followed by the addition of 7.5 mL of ACN. The mixture was then transferred into a 50 mL Falcon tube containing the extraction salts from the Phenomenex roQ QuEChERS Extraction Kit (KS0‐8909), which includes 4.0 g MgSO_4_, 1.0 g NaCl, 1.0 g sodium citrate dihydrate (SCTD), and 0.5 g sodium citrate sesquihydrate (SCDS). The sample was vortexed vigorously for 1 min and then centrifuged 224 × g for 5 min. For the clean‐up step, 5 mL of the supernatant were transferred to a 15 mL tube containing the Phenomenex dSPE clean‐up kit (KS0‐9507), which consists of 900 mg MgSO_4_ and 150 mg primary‐secondary amine (PSA). The mixture was again vortexed for 30 s, followed by centrifugation at 4000 rpm for 5 min to separate the solid phase. The resulting supernatant was transferred into an auto sampler vial for subsequent UHPLC‐DAD analysis. Addition/recovery assays were performed to ensure satisfactory results by spiking the samples with 25 mg L^−1^ of each compound.

## 3 Results and Discussion

### 3.1 Chromatographic Separation

Chromatographic separation was optimized through a Box–Behnken DoE. The matrix of independent variables, i.e., gradient time and temperature, and the corresponding responses, i.e., resolution, retention time, and theoretical plate numbers, is presented in Table S2. Three‐dimensional response surface plots were generated to assess the effects of gradient time and column temperature on key chromatographic performance parameters (Figures S3 and S4). Second‐degree polynomial regression models were fitted to describe the effects of both studied variables on key chromatographic responses (Table S3).

Optimal chromatographic performance was achieved at a gradient time of 11.5 min and a column temperature of 30°C, conditions for which the DoE model predicted an *Rs_min_* of 6.66 and >95,000 theoretical plates for AZO. Experimental validation under these conditions resulted in *Rs_min_* values ranging from 3.67–6.13 (Table S2), demonstrating good agreement with the model predictions and confirming that the selected parameters ensured baseline separation of all analytes.

The response surface plots (Figure S3) showed a pronounced curvature for most of the fitted surfaces, indicating that the interaction between gradient time and column temperature was statistically significant. Figure S3a illustrates that an increase in column temperature resulted in a noticeable decrease in resolution (*Rs_min_*), especially when combined with a shorter gradient time. This behavior can be attributed to the reduction in mobile‐phase viscosity and the weakened analyte–stationary‐phase interactions at elevated temperatures, which accelerate elution but reduce chromatographic selectivity. Conversely, longer gradient times improved resolution by allowing better separation of closely eluting compounds, although excessively long gradients caused slight peak broadening and increased the overall analysis time. As illustrated in Figures S3b and S3c, both retention time and theoretical plate number were also influenced by the same variables: increasing temperature shortened retention and slightly reduced column efficiency, whereas extending the gradient enhanced peak capacity. These findings confirm that the optimized conditions represent the best compromise among resolution, efficiency, and analysis time. To further evaluate the separation performance, response surface plots were generated to examine the effect of gradient time and column temperature on the resolution between adjacent analyte peaks (Figure S4). Distinct resolution trends were observed for each compound pair, allowing the identification of optimal separation regions and supporting the robustness of the selected chromatographic conditions.

Thus, the most effective chromatographic conditions were achieved using a KinetexXB‐C18 column and a gradient elution mode composed of a mobile phase of MeOH with 0.1% formic acid (A) and 0.1% formic acid in water; and (B), pH 3.35, with the gradient time summarized in Table S4.

Although analytes were separated within approximately 13 min, the gradient was extended to 15 min to allow full column re‐equilibration. Shorter gradients or lower temperatures resulted in reduced resolution, while longer runs or higher temperatures did not produce any significant improvement. Chromatographic parameters, such as retention time, peak area, resolution, peak symmetry, and number of theoretical plates are summarized in Table S5. A representative chromatogram under optimized conditions for increasing analyte concentrations (3.75–100 mg L^−1^) of the five target pesticides is presented in Figure S5. An injection volume of 1 µL was used.

### 3.2 Selection of SPE Resin and Extraction Variables Optimization

#### 3.2.1 Sorbent Performance

**FIGURE 2:**
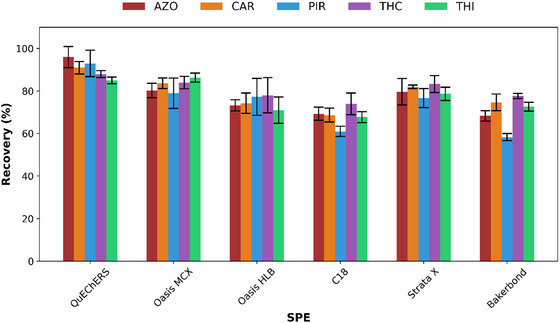
Recoveries (%) of target pesticides obtained using different extraction methods: conventional QuEChERS method and 3D‐printed SPE devices functionalized with various sorbents (Oasis MCX, Oasis HLB, C18, Strata X, and Bakerbond) prior to HPLC‐DAD analysis. Bars represent mean values ± SD (*n* = 3). AZO: azoxystrobin, CAR: carbaryl, PIR: pirimicarb, THC: thiacloprid, THI: thiamethoxam.

The 3D‐printed devices were coated using the “stick and cure” technique with various SPE sorbents, including Oasis MCX, Oasis HLB, C18, Strata‐X, and Bakerbond, to evaluate their retention and recovery efficiencies for the five target pesticides. The performance of each sorbent was assessed under the extraction conditions recommended by the manufacturers using spiked honey samples. In order to compare the recoveries obtained with different 3D‐printed coated devices for the pesticide extraction, a QuEChERS extraction followed by UHPLC‐DAD analysis was also performed. As shown in Figure 2, the highest recoveries for all pesticides were obtained with QuEChERS dSPE (85–96 %), while the best results for the 3D‐printed coated device were achieved with Oasis MCX (79–86 %). The QuEChERS’ high recovery rates were likely attributable to the dispersive nature of the extraction, which involves agitation that enhances mass transfer. The 3D‐printed devices coated with Strata X (76–83 %), Oasis HLB (71–78 %), C18 (61–74 %), and Bakerbond (58–77 %) resins exhibited lower recoveries compared with Oasis MCX (82–98%).

Among the tested materials, Oasis MCX exhibited the highest and most reproducible recoveries for all analytes. Its mixed‐mode cation‐exchange mechanism, which combines hydrophobic and ionic interactions, enhanced both retention and selectivity for compounds bearing basic or moderately polar functional groups (e.g., PIR and THC). In contrast, other resins such as C18 and HLB showed lower recoveries and less efficient matrix clean‐up. Therefore, Oasis MCX was selected as the most suitable sorbent for further optimization and validation.

The extraction mechanism between the target pesticides and the Oasis MCX sorbent primarily involves a combination of hydrophobic, π–π, and ionic interactions. The MCX material contains sulfonic acid functional groups, which act as a strong cation exchanger and promote electrostatic attraction with basic nitrogen‐containing analytes such as PIR, THC, and THI. In addition, the polymeric backbone contributes to hydrophobic interactions that enhance the retention of moderately polar pesticides such as AZO and CAR. This dual retention mechanism enhances both selectivity and recovery efficiency for the multiclass analytes investigated.

#### 3.2.2 Statistical Comparison

ANOVA comparing the mean recoveries across different SPE sorbents (QuEChERS, Oasis MCX, Oasis HLB, C18, Strata X, and Bakerbond) revealed a statistically significant difference between groups (*p* < 0.001). This finding confirms that the type of SPE sorbent has a significant influence on extraction efficiency. The observed differences in recovery rates among the tested SPE sorbents were mainly attributable to the lipophilicity of the target analytes and the physicochemical properties of the sorbent materials. More lipophilic compounds, such as AZO and CAR, which are characterized by high log P values, exhibited stronger interactions with non‐polar polymeric sorbents, such as Strata X and Oasis MCX, resulting in improved retention and cleaner extracts, particularly in complex matrices such as honey. In contrast, conventional silica‐based sorbents (e.g., C18 and Bakerbond) rely primarily on hydrophobic interactions to achieve reversed‐phase retention. However, their selectivity is markedly reduced in complex matrices and in multi‐residue analyses involving analytes spanning a wide polarity range. This limitation accounts for their lower performance compared with more advanced polymeric materials.

**FIGURE 3:**
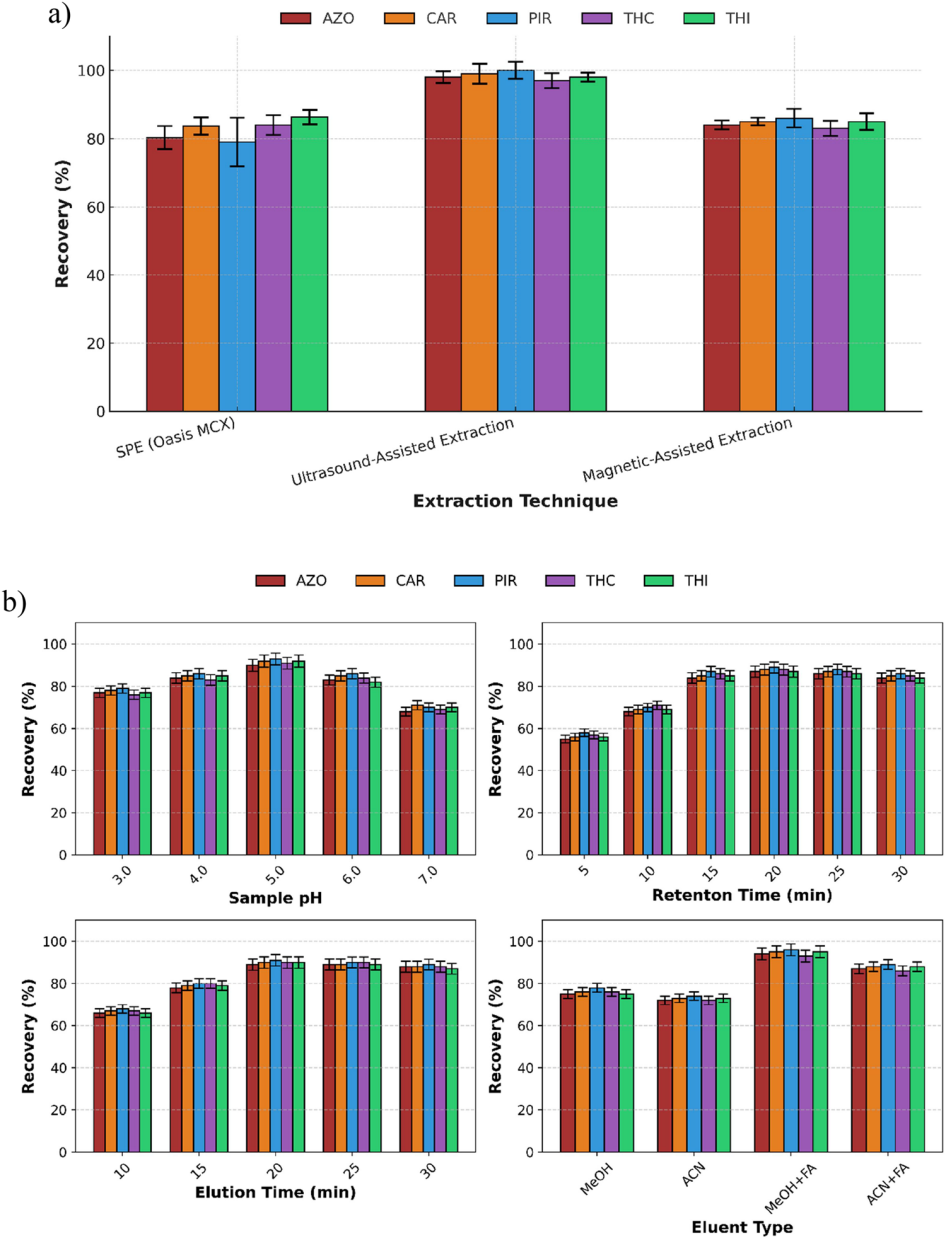
Recoveries (%) of target pesticides obtained using 3D‐printed device coated with Oasis MCX under different extraction conditions (a) without assistance, with ultrasound‐ and magnetic stirring‐ assisted extraction (retention and elution times of 20 min); and (b) varying sample pH, retention and elution times. Bars represent mean values ± SD (*n* = 3). AZO: azoxystrobin, CAR: carbaryl, PIR: pirimicarb, THC: thiacloprid, THI: thiamethoxam.

Oasis MCX, a mixed‐mode sorbent that combines reversed‐phase and cation‐exchange functionalities, showed consistent performance across all target compounds. This performance was mainly attributed to its dual retention mechanisms, which enable the effective retention of both neutral and slightly basic pesticides, such as THI and THC. The Tukey HSD post‐hoc grouping (Figure S6) placed Oasis MCX in an intermediate statistical group (ab), with recovery values closely aligned with those of the highest‐performing reference material. Consequently, Oasis MCX was selected for coating the 3D‐printed devices, and two extraction techniques were evaluated, namely magnetic stirring and UAE. Although both methods provided satisfactory results, UAE showed superior performance in terms of analyte recovery (96–100 %), representing an improvement of approximately 10–20% compared with the unassisted extraction, and was therefore selected for further use (Figure 3a). Thus, UAE was integrated into the proposed methodology to enhance analyte transfer from honey matrix. The ultrasonic bath was operated at a power of 180 W and a frequency of 42 kHz. Compared with conventional magnetic stirring, UAE provided a more efficient approach to enhancing mass transfer and increasing analyte recovery. The application of ultrasound induces cavitation, which promotes more effective interactions between the sample matrix and the extraction solvent, thereby resulting in higher extraction efficiency. These advantages are particularly relevant for complex food matrices such as honey, where efficient analyte release is critical.

#### 3.2.3 Extraction Variable Optimization

Then, several critical parameters were studied by univariate approach to obtain the optimal extraction performance, i.e., sample pH, retention and elution times, and eluent composition. As shown in Figure 3b, the highest recoveries were achieved at pH 5.0. Since the recoveries slightly decreased at pH 4 and 6, a pH adjustment of 5 ± 0.5 was selected for subsequent experiments.

Extraction efficiency was strongly influenced by sample pH, with the highest recoveries obtained at pH 5.0. The Oasis MCX resin contains cation‐exchange sites bonded to a reversed‐phase matrix, providing dual retention mechanisms. At pH 5, the sulfonic acid groups of the sorbent remain fully ionized, while basic nitrogen atoms in several target pesticides (e.g., PIR, THC, THI) are partially protonated. This promotes strong electrostatic interactions, while hydrophobic forces further retain less polar compounds such as CAR and AZO. At lower pH (< 4), excessive protonation reduces hydrophobic affinity, whereas at higher pH (> 6), decreased protonation weakens cation‐exchange interactions. Consequently, pH 5.0 represents an optimal balance of ionic and hydrophobic interactions, yielding the highest recoveries.

For the extraction step, a one‐variable‐at‐a‐time (OVAT) approach was used to simplify the evaluation of categorical factors such as elution solvent type and extraction technique.

Because ACN was used as the desorption solvent, NaCl was added to promote phase separation by reducing ACN–water miscibility and facilitating pesticide partitioning into the organic phase. To assess this effect, three assays were performed using 1.0, 1.5, and 2.0 g NaCl. As recoveries did not differ significantly, the minimum amount (1.0 g) was selected (Figure S7). This reduced salt content ensured effective separation within the honey matrix while minimizing salt‐related issues often observed in conventional QuEChERS workflows.

Then, retention and elution times for the UAE were evaluated over a range of 5–30 min. The highest recoveries were achieved at extraction times of 20 min or longer. Therefore, both retention and elution times were set at 20 min, which enhanced sorbent–analyte interaction by increasing mass transfer and promoting more efficient adsorption. The combined use of vortex mixing and ultrasonic treatment during both the retention and elution stages significantly enhanced mass transfer processes between the sample matrix and the sorbent surface. Specifically, vortex agitation (1 min) followed by sonication (20 min) during the retention step improved analyte adsorption onto the sorbent by facilitating dispersion and penetration of the sample through the coating. Applying the same sequence during the elution step promoted efficient desorption of the retained analytes, resulting in higher and more reproducible recovery rates. Overall, this synergistic approach contributed to a more robust and efficient extraction process.

To identify the most effective desorption solvent, MeOH and ACN were each tested with and without 0.1% formic acid. Among these, MeOH containing 0.1% formic acid consistently yielded the highest recoveries across all pesticides and was therefore selected as the optimal desorption solvent due to its superior recovery efficiency (Figure 3b). The addition of formic acid likely enhanced analyte desorption by weakening interactions between the sorbent and the retained compounds. MeOH alone was used to precondition the resin forming the coating of the 3D‐printed device, ensuring proper sorbent activation and thereby enhancing analyte retention.

#### 3.2.4 Reusability Assessment

**FIGURE 4:**
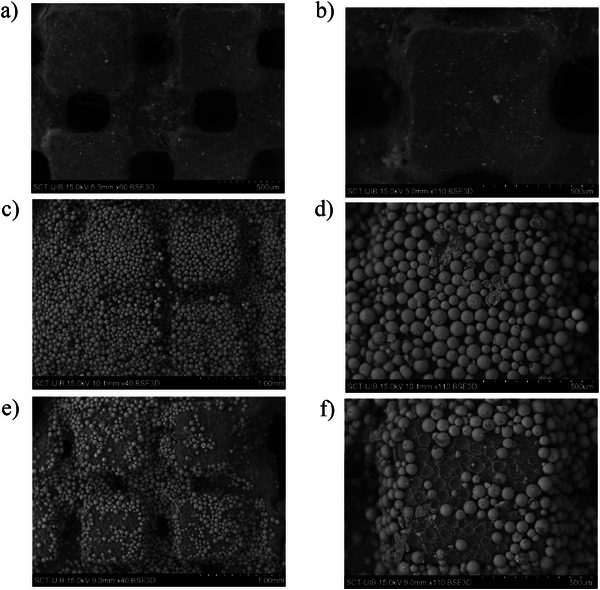
SEM images illustrating the surface morphology of the 3D‐printed SPE device at different stages of use: (a) x 60 and (b) x 110 uncoated device images; (c) x 40 and (d) x 110 coated device with Oasis MCX (prior to UAE); (e) x 40 and (f) x 110 coated device after UAE.

Finally, the reusability of the 3D‐printed coated device was assessed over two consecutive extraction cycles. Despite recoveries remaining within acceptable limits (Table S6), signs of polymer fatigue and partial delamination of the sorbent layer were observed after the second use. SEM images of the 3D‐printed device with and without the Oasis MCX coating before and after use (two cycles) are shown in Figure 4. These results indicate limited mechanical durability under repeated operation. However, the environmental advantages of the proposed approach mainly arise from its miniaturized design, reduced solvent and sample consumption, and elimination of single‐use SPE cartridges, rather than from repeated reuse of the device. Thus, to ensure analytical reliability, device reuse is not recommended or should be limited to a maximum of two cycles.

### 3.3 UAE 3D‐Printed Coated Device UHPLC‐DAD Method Validation

**FIGURE 5:**
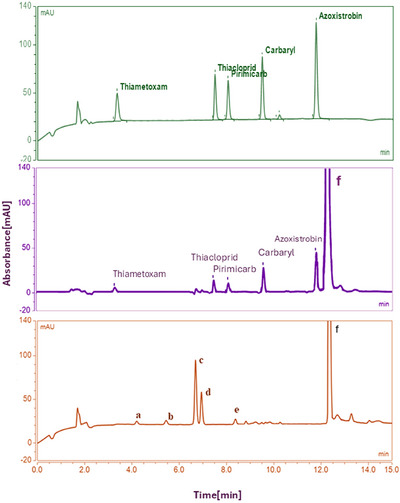
Representative chromatograms (UHPLC‐DAD) of a pesticide standard mixture (green, 50 mg L^−1^ each; no extraction), and a honey sample spiked with the target analytes (purple, 25 mg L^−1^ each), and a blank honey extract (orange), both obtained using the 3D‐printed SPE‐coated device (with blank subtraction). Operative conditions: Kinetex XB‐C18 column, 100 × 3 mm, 2.6 µm; mobile phase: methanol (A) and 0.1% formic acid in water (B), pH 3.35; gradient time: 11.5 min; column temperature: 30°C; flow rate: 0.25 mL min^−1^; injection volume: 1 µL. Peaks a–f: unknown compounds.

Figure 5 shows a representative chromatogram obtained using the developed UHPLC‐DAD method, including a pesticide standard for peak visualization (without extraction procedure), a blank honey and a spiked honey sample, both analyzed with the UAE 3D‐printed coated device.

Analyte identification was performed by comparing retention times with authentic standards and confirming spectral profiles using diode‐array detection (200–400 nm). Additionally, spiked honey samples yielded chromatographic and spectral characteristics identical to those of the reference standards.

Reagent blanks and blank honey extraction controls were performed using the 3D‐printed device under the same experimental conditions but without the addition of pesticides. The resulting chromatograms showed no detectable peaks at the retention times corresponding to the target analytes, confirming that neither the 3D‐printed material nor the Oasis MCX coating contributed any contaminants to the extracts. Meanwhile, spiked samples showed well‐resolved, baseline‐separated peaks with consistent retention times and recoveries. These findings demonstrate that the proposed method exhibits selectivity for the analytes of interest under the optimized chromatographic conditions.

As can be observed, besides the target analytes' peaks, there are two peaks at the end of the chromatogram, which correspond to unknown substances possibly leached from the 3D‐printed device, as they appear in the reagent blank chromatograms at the same retention times. Since the AZO peak exhibited good *Rs_min_* when injecting 1 µL of eluate, no changes were made to the previously optimized chromatographic method.

MLODs and MLOQs were established from matrix‐matched calibration data using signal‐to‐noise (S/N) ratios of 3:1 and 10:1, respectively. The S/N ratios were calculated based on chromatographic peaks at the lowest detectable concentration of the matrix‐matched standards. Each concentration level was analyzed in triplicate to confirm reproducibility of the detection limits. LODs ranged from 0.001 to 0.1 mg kg^−1^, while LOQs ranged from 0.004 to 0.4 mg kg^−1^ and were expressed in mg kg^−1^, in accordance with EU Regulation (EC) No 396/2005 for pesticide residues in honey. The obtained LOQs (0.004–0.4 mg kg^−1^) are of the same order of magnitude as the default MRL of 0.05 mg kg^−1^ established for honey. Although some values slightly exceed this limit, MRLs define legal tolerance thresholds rather than analytical performance. Therefore, the achieved sensitivity was considered sufficient for monitoring purposes using UHPLC–DAD, particularly considering that the primary aim of this study was to validate the 3D‐printed extraction platform, which can be easily adapted to LC–MS/MS for ultra‐trace (ng g^−1^) level confirmation.

The method demonstrated good linearity, with determination coefficients (*R^2^*) >0.99 for all target pesticides across the concentration range of 0.05–100 mg L^−1^ (Figure S2). Precision values below than 5.1 % RSD were obtained for all pesticides. A PF of 6.5 was achieved, meanwhile the EFs ranging from 4.0 to 4.8 were calculated, depending on the analyte.

Matrix effects values ranged from –9.2% to + 4.1%, with most pesticides showing minimal signal suppression (<15%). These low deviations indicate that the proposed methodology provided effective cleanup and selective extraction of the target analytes, minimizing the co‐extraction of interfering matrix components. The negligible suppression or enhancement observed confirms the suitability of the method for quantitative analysis of honey samples without the need for additional correction factors.

The figures of merit of the developed UAE–3D‐printed coated device‐UHPLC‐DAD method are summarized in Table 1.

**TABLE 1:** Figures of merit of the ultrasound assisted 3D‐printed coated device extraction method followed by UHPLC‐DAD analysis. AZO: Azoxystrobin, CAR: Carbaryl, PIR: Pirimicarb, THC: Thiacloprid, THI: Thiamethoxam.

Parameter	AZO	PIR	CAR	THC	THI
Linearity range (mg L^−1^)*	0.14–100	0.005–100	1.6–100	0.4–100	0.5–100
*R^2^*	0.993	0.994	0.990	0.993	0.987
LOD (mg kg^−1^)	0.03	0.001	0.04	0.1	0.1
LOQ (mg kg^−1^)	0.1	0.004	0.1	0.3	0.4
Precision Intra‐day precision (RSD %, *n* = 3)	3.4	5.1	4.6	3.8	4.2
Inter‐day precision (RSD %, *n* = 9)	4.9	6.7	6.2	5.5	6.0
Recovery (%)	93–105	96–108	90–107	95–109	97–104
Preconcentration factor (PF)	6.5	6.5	6.5	6.5	6.5
Enrichment factor (EF)	4.2	4.6	4.0	4.5	4.8
Matrix effect	−6.5	−9.2	4.1	−5.8	−3.3

*Note*: *standard concentration range.

The use of a 3D‐printed structure as a support for the MCX resin provides several advantages over the use of the sorbent alone in a conventional cartridge format. The stereolithographically fabricated geometry offers a controlled and reproducible surface for uniform sorbent coating, ensuring consistent extraction performance across devices. Furthermore, the open porous architecture of the 3D structure enhances mass transfer and reduces solvent and sample consumption by promoting efficient fluid circulation through the coated surface. Compared with direct use of Oasis MCX cartridges, the 3D‐printed device achieved comparable or even higher recoveries (89–106%) with reduced solvent volumes and partial reusability.

Finally, the analysis of real honey samples (collected in Mallorca, Spain) was carried out employing the developed extraction methodology, and addition/recovery tests were performed at three fortification levels (Table 2). The standard addition levels (31.5, 56.5, and 81.5 mg L^−1^) were selected within the method's linear range to evaluate recovery and repeatability rather than to match regulatory MRL values, since the purpose of the study was methodological development. All detected pesticide residues were below the LOQs, indicating the absence of quantifiable contamination. The samples were then spiked with a mixed pesticide standard solution to reach final concentrations ranging from 31.5 to 100 mg L^−1^. The method yielded recoveries ranging from 90 to 109 %, confirming its accuracy and reliability for the analysis of pesticide residues in honey. A one‐way ANOVA was conducted to compare recoveries of each pesticide across the three fortification levels. The results showed no statistically significant differences (*p* > 0.05) for any compound, indicating that recovery performance remained consistent regardless of concentration levels. This confirms the robustness and reproducibility of the developed UAE‐3D‐printed extraction method across the studied range (Table S7). Future work should involve applying the developed 3D‐printed extraction platform to a larger and more diverse set of commercial honey samples to further confirm its robustness and applicability to different botanical and geographical origins.

**TABLE 2:** Analysis of real honey samples and corresponding addition/recovery tests. R: recovery, RSD: relative standard deviation. AZO: azoxystrobin, CAR: carbaryl, PIR: pirimicarb, THC: thiacloprid, THI: thiamethoxam.

Pesticide	—	Low level			—	Medium level			—	High level		
	Added (mg L^−1^)	Found (mg L^−1^)	R (%)	RSD (%)	Added (mg L^−1^)	Found (mg L^−1^)	R (%)	RSD (%)	Added (mg L^−1^)	Found (mg L^−1^)	R (%)	RSD (%)
AZO	0	<LOD	—	—	0	<LOD	—	—	0	<LOD	—	—
	31.5	33.0	105	1.6	56.5	54.6	97	2.6	81.5	85.0	104	1.6
	37.5	38.6	103	3.3	62.5	61.3	98	1.6	87.5	89.0	102	1.7
	50.0	49.5	99	2.6	75.0	70.0	93	1.2	100.0	98.3	98	1.0
PIR	0	<LOD	—	—	0	<LOD	—	—	0	<LOD	—	—
	31.5	34.0	108	1.7	56.5	57.0	101	3.5	81.5	82.0	101	2.6
	37.5	39.5	105	2.0	62.5	63.0	101	1.8	87.5	90.0	103	1.1
	50.0	48.0	96	0.4	75.0	78.0	104	2.8	100.0	96.0	96	1.7
CAR	0	<LOD	—	—	0	<LOD	—	—	0	<LOD	—	—
	31.5	30.5	97	0.6	56.5	55.2	98	0.5	81.5	83.5	102	2.8
	37.5	39.5	105	2.5	62.5	56.3	90	1.5	87.5	94.0	107	1.4
	50.0	48.0	96	1.3	75.0	77.0	103	3.5	100.0	99.0	99	1.0
THC	0	<LOD	—	—	0	<LOD	—	—	0	<LOD	—	—
	31.5	30.0	95	0.9	56.5	55.0	97	1.7	81.5	82.0	101	1.6
	37.5	39.5	105	0.3	62.5	63.9	102	1.6	87.5	95.0	109	0.7
	50.0	49.0	98	1.1	75.0	77.2	103	1.7	100.0	104.3	104	1.6
THI	0	<LOD	—	—	0	<LOD	—	—	0	<LOD	—	—
	31.5	31.4	99	0.2	56.5	58.8	104	2.6	81.5	80.0	98	1.0
	37.5	36.4	97	4.8	62.5	64.3	103	2.7	87.5	88.5	101	1.2
	50.0	52.0	104	1.6	75.0	76.2	102	1.0	100.0	103.0	103	2.0

*Note*: LOD: limit of detection.

### 3.4 Greenness of the Proposed Methodology

**FIGURE 6:**
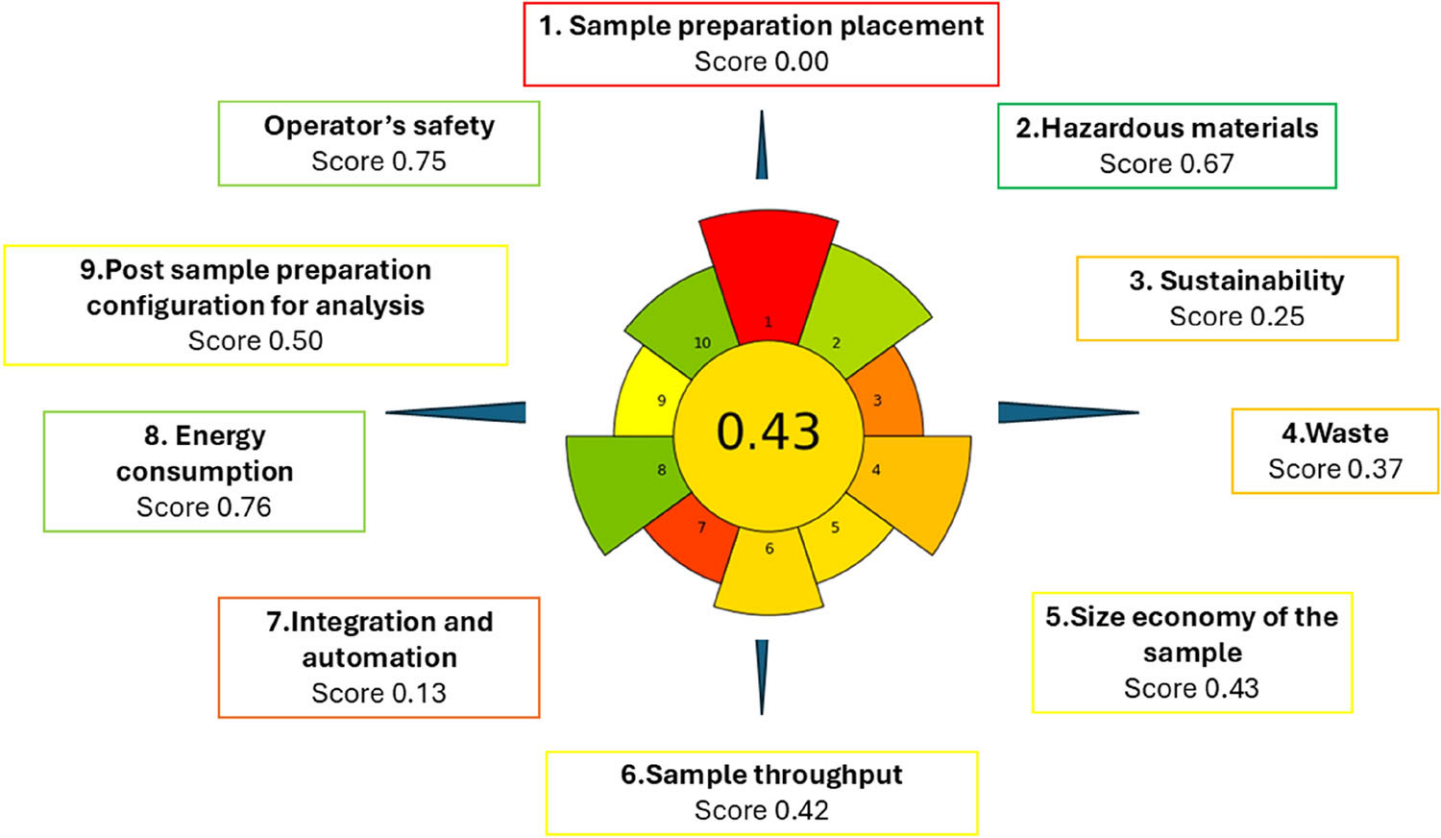
Greenness assessment of the ultrasound assisted 3D‐printed coated device extraction method using the AGREEprep metric.

The environmental impact of the proposed methodology was evaluated using the AGREEprep tool, which quantitatively assesses ten criteria, namely: sample preparation position, use of hazardous materials, sustainability, waste generation, sample amount, sampling frequency, degree of automation, energy consumption, post preparation treatment, and analyst safety. The greenness assessment of the proposed extraction protocol yielded an overall AGREEprep score of 0.43, indicating a moderately green sample preparation approach (Figure 6). This score reflects a balance between environmental sustainability, operational simplicity, and analytical performance. The lowest value (0.00) corresponded to sample preparation positioning due to the ex‐situ and manual nature of the extraction. Favorable scoring for hazardous materials (0.67) resulted from the use of small amounts of ACN and MeOH and the absence of halogenated or highly toxic solvents. The sustainability, renewability, and reusability criteria (0.25) reflect the partial reusability of the 3D‐printed SPE device (1–2 cycles) and the limited renewable origin of the reagents. SEM analysis revealed early polymer fatigue and partial coating delamination after two cycles, indicating limited mechanical stability but adequate performance within this reuse range.

Waste generation (0.37) and sample amount economy (0.43) were influenced by moderate solvent volumes (∼9 mL per sample) and the reduced sample mass (5 g), lowering overall consumption relative to conventional approaches. Sample throughput (0.42) was moderate (≈ six samples per hour) and primarily limited by manual handling, while integration and automation received the lowest score (0.13), typical of prototype‐stage 3D‐printed systems. Conversely, energy consumption obtained one of the highest scores (0.76) due to the use of low‐power UAE. The post‐sample preparation configuration (0.50) corresponds to UHPLC‐DAD analysis, which provides reliable quantification with moderate solvent use and without requiring mass spectrometry. Operator safety also scored highly (0.75) owing to small solvent volumes and low‐temperature handling conditions that minimize exposure.

To frame the environmental relevance of our findings, we compared them with the conventional QuEChERS procedure commonly used for pesticide analysis in honey. Ravi et al. recently evaluated several extraction strategies for pesticides and biopesticides in honey and reported an AGREEprep score of 0.34 for a standard QuEChERS workflow (Ravi et al. 2025). This relatively low value reflects the inherent limitations of traditional QuEChERS, particularly its substantial reliance on ACN, salting‐out agents, and dispersive sorbents—constraints also highlighted in previous evaluations (Almeida et al. 2020). In our study, the AGREEprep assessment yielded a score of 0.43, indicating that the proposed method provides a moderately greener alternative to the conventional QuEChERS approach in terms of sample‐preparation sustainability.

Unlike conventional QuEChERS workflows which require mixed salts (e.g., MgSO_4_, NaCl, buffering agents) to induce phase separation, the present method relies solely on NaCl. This single‐salt strategy proved sufficient for phase separation in the honey matrix while reducing residual ionic content and matrix interferences, thereby improving greenness and operational simplicity. A side‐by‐side comparison of the evaluated methodologies can be found in Table S8.

Overall, the AGREEprep evaluation confirms that the ultrasound‐assisted 3D‐printed extraction approach provides a greener, simpler, miniaturized, and energy‐efficient alternative to QuEChERS‐type procedures. Future developments will focus on greener solvent systems, increased automation, and improved material durability.

## 4 Conclusions

The integration of UAE with a 3D‐printed device coated with SPE resin enabled effective multi‐residue analysis of pesticides in honey using UHPLC‐DAD. The method demonstrated good sensitivity, precision, and accuracy. Its application to real honey samples confirmed its suitability for trace‐level determinations, with all samples showing pesticide concentrations below the LOQ values.

Chromatographic separation was optimized using a Box–Behnken DoE. The final method achieved resolution values higher than 6.6 for all analyte pairs, under the following conditions: a gradient time of 11.5 min, a column temperature of 30°C, and a mobile phase consisting of MeOH and 0.1% formic acid at pH 3.35.

Among the tested SPE sorbents, Oasis MCX was selected as it provided the highest recoveries and was used for coating via the “stick and cure” technique. The integration of ultrasound into the extraction protocol, further improved reliability and reproducibility, confirming its suitability for pesticide residue analysis.

Once the proposed methodology was optimized, high recoveries comparable to those obtained with the QuEChERS method were achieved. Thus, the proposed methodology offers several notable advantages, including customizability, minimal SPE resin, solvent, and salt consumption and operational simplicity, as the resin does not need to be recovered as in dispersive extraction. Besides, its miniaturized, customizable design allows low‐cost, on‐demand production and can be readily adapted to other analytes or matrices, representing a promising step toward greener and more sustainable sample preparation in pesticide residue analysis.

This study highlights the potential of 3D‐printed extraction coated devices as modular, cost‐effective, and sustainable platforms for pesticide residue analysis. The proposed approach is easily adaptable to other analyte classes and food matrices, offering a promising alternative to conventional extraction techniques.

## Author Contributions

**Daniela Lupu**: investigation, validation, formal analysis, writing – original draft, software. **Gabriel Hancu**: conceptualization, methodology, writing – review and editing, funding acquisition, project administration. **Laura Ferrer**: conceptualization, supervision, resources, writing – review and editing, data curation, funding acquisition.

## Conflicts of Interest

The authors declare no conflicts of interest.

## Supporting information




**Supplementary Material**: jfds70788‐sup‐0001‐SuppMat.docx
