# Supplementary material for: Ultrasound‐Assisted Extraction Using a 3D‐Printed Device Functionalized With SPE Sorbent for UHPLC‐DAD Analysis of Pesticide Residues in Honey
Source: J Food Sci. 2025 Dec 28;91(1):e70788. doi: 10.1111/1750-3841.70788 (PMC12745918; doi:10.1111/1750-3841.70788)
Supplement: Supplementary file 1 — Supplementary Material: jfds70788‐sup‐0001‐SuppMat.docx [file JFDS-91-0-s001.docx]

**Ultrasound-assisted extraction using a 3D-printed device functionalized with SPE sorbent for UHPLC-DAD analysis of pesticide residues in honey**

Daniela Lupu^a,b^, Gabriel Hancu^b^, Laura Ferrer^a^ *

*^a^Environmental Analytical Chemistry Group, University of the Balearic Islands, Cra. Valldemossa km. 7.5, 07122 Palma, Spain.*

*^b^ Department of Pharmaceutical and Therapeutic Chemistry, Faculty of Pharmacy, “George Emil Palade” University of Medicine, Pharmacy, Science and Technology of Târgu Mures, Gh. Marinescu 38, 540139 Târgu Mures, Romania*

**Corresponding author:* [*laura.ferrer@uib.es*](mailto:laura.ferrer@uib.es)

**Supplementary material**

**
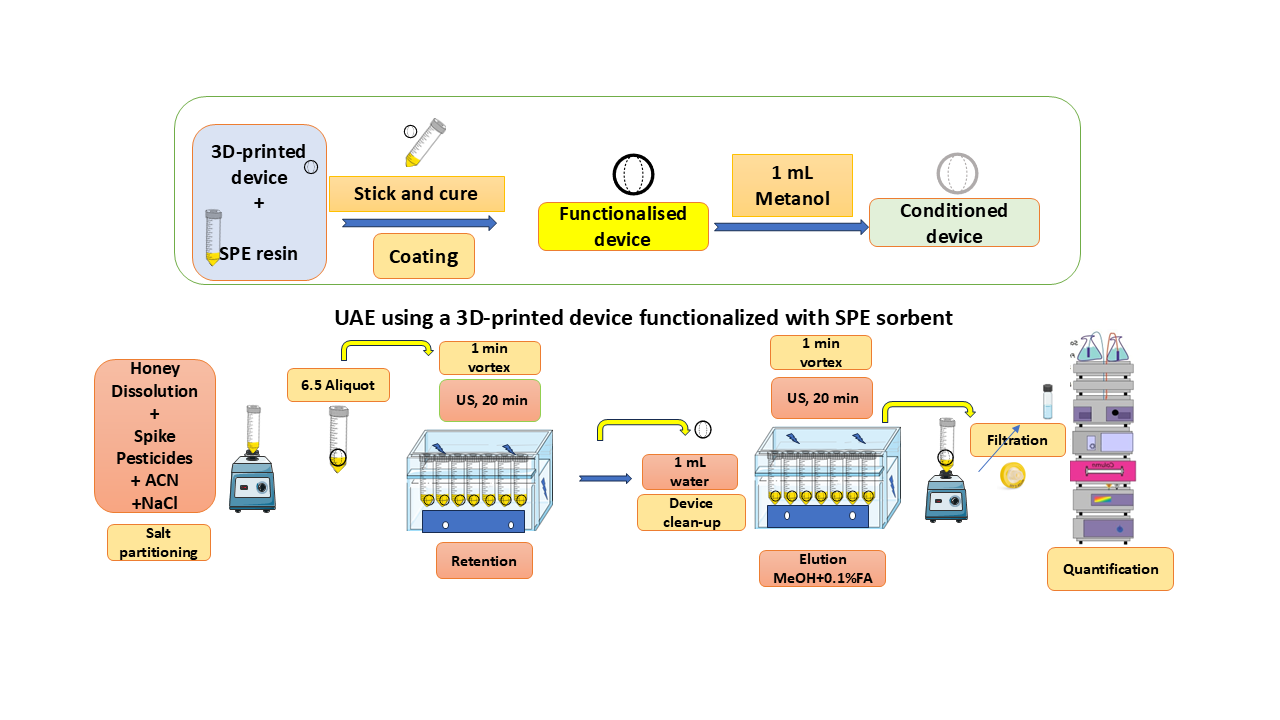
**

**Figure S1.** Sample preparation workflow exploiting the developed ultrasound-assisted extraction (UAE) method based on 3D-printed SPE device coated with MCX sorbent.

**a)**

**
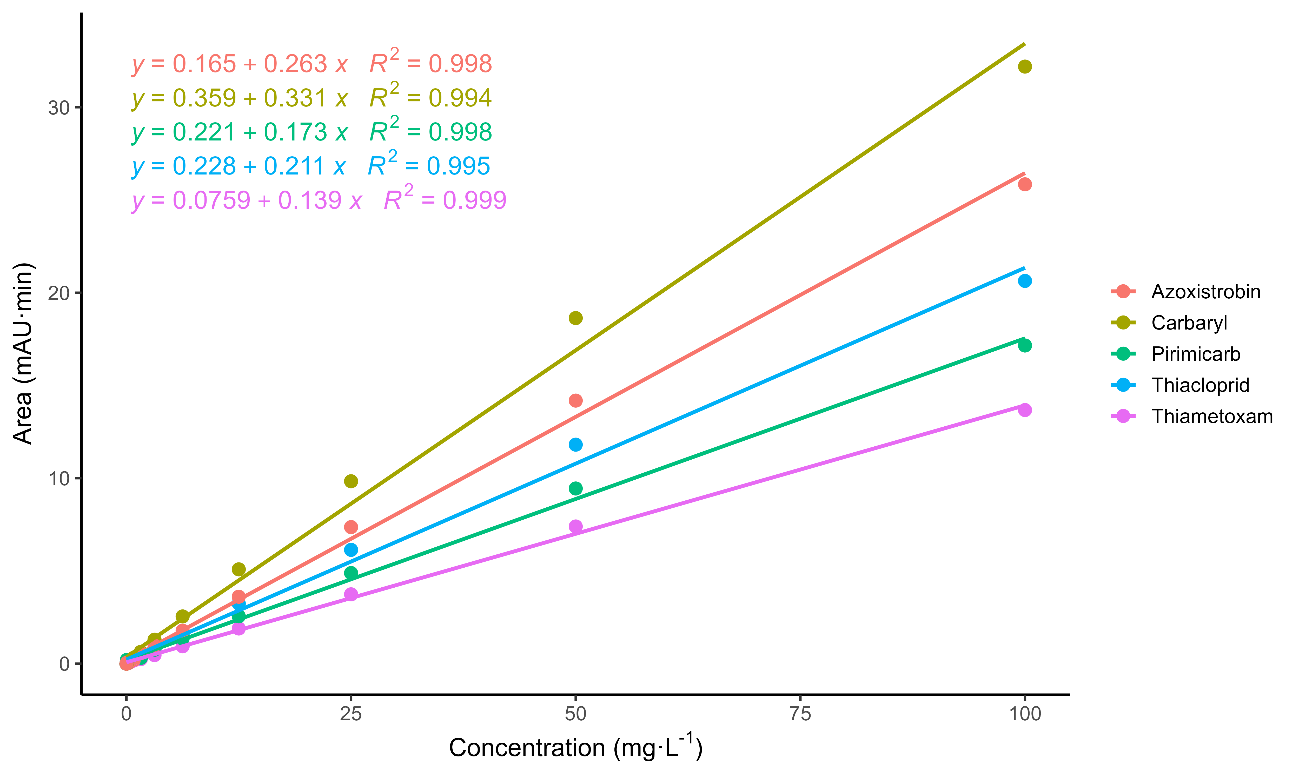
**

**b)**

**
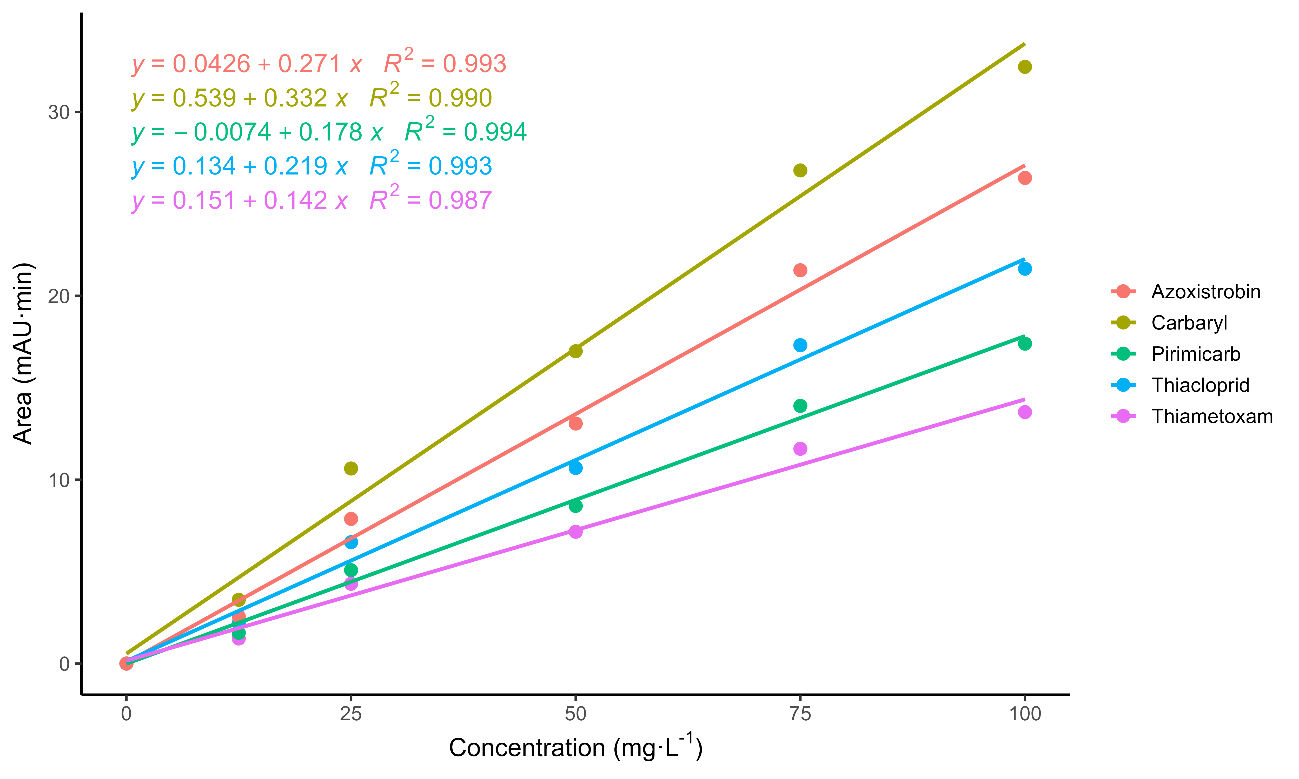
**

**Figure S2.** Solvent-based (a) and matrix-matched (b) calibration curves for the quantification of pesticides using the UAE–3D-printed coated device followed by UHPLC-DAD, constructed over the concentration range of 0.05–100 mg L⁻¹.

a)

b)

c)

**
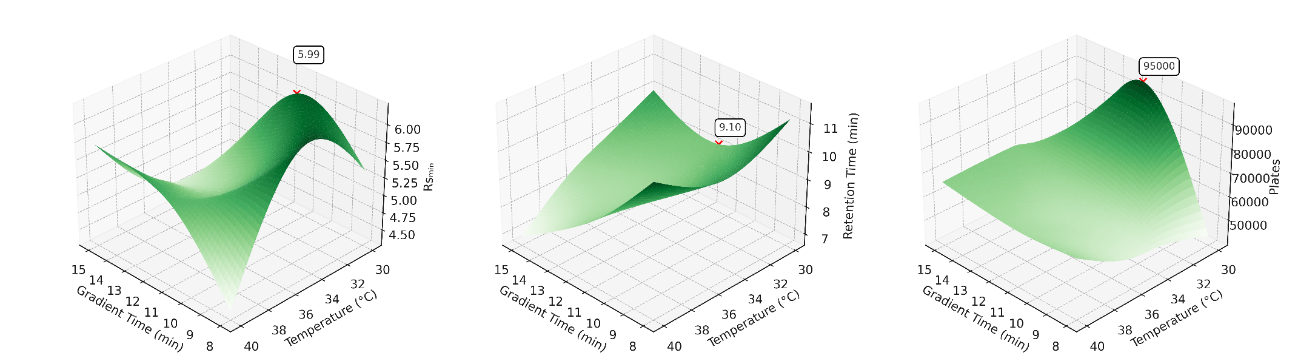
**

**Figure S3.** Response surface plots illustrating the effect of gradient time and column temperature on key chromatographic parameters: (a) minimum resolution (Rsₘᵢₙ) across adjacent peak pairs, (b) average retention time of the five analytes, and (c) theoretical plate number determined using the peak of Azoxystrobin.

**
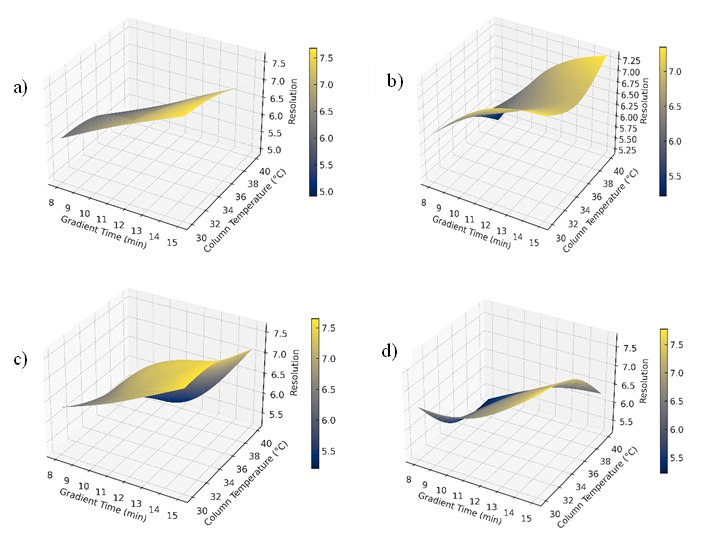
**

**Figure S4**. Response surface plots illustrating the effect of gradient time and column temperature on the chromatographic resolution (Rs) between adjacent analyte peaks. The pattern reflects consistent chromatographic behavior within the system (a) Pirimicarb–Carbaryl: Rs increases with longer gradient times but may slightly decline at higher temperatures; (b) Carbaryl–Azoxystrobin: Rs improves significantly with both gradient time and temperature, showing strong sensitivity to both variables; (c) Thiamethoxam–Thiacloprid: optimal RS is achieved at intermediate gradient times and temperatures, indicating a balanced response; (d) Thiacloprid–Pirimicarb: RS exhibits a complex, non-monotonic response with two distinct optimal regions, one at high gradient time and low temperature, and another at moderate gradient time and temperature.

**
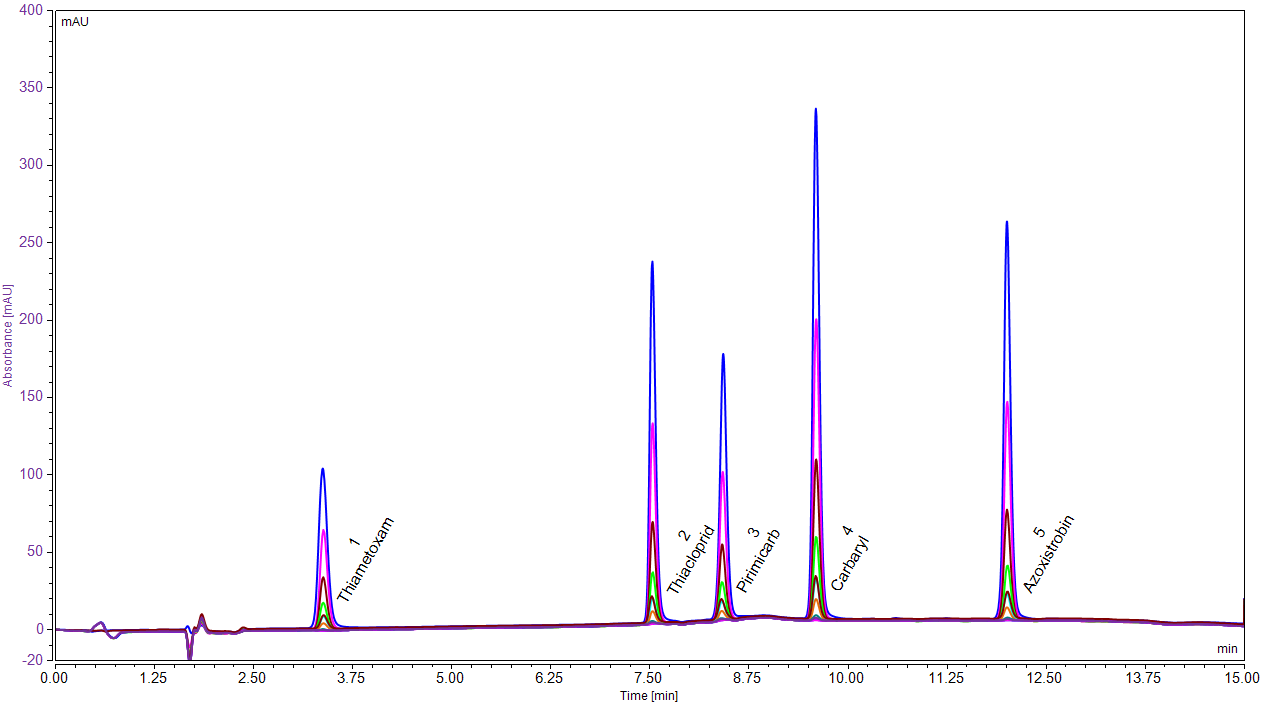
**

**–––––– 100 mg L⁻¹**

**–––––– 75 mg L⁻¹**

**–––––– 50 mg L⁻¹**

**–––––– 25 mg L⁻¹**

**–––––– 12.5 mg L⁻¹**

**–––––– 6.25 mg L⁻¹**

**–––––– 3.75 mg L⁻¹**

**Figure S5.** Chromatographic separation of five pesticides via UHPLC-DAD (without extraction procedure), for increasing concentrations of standards (3.75-100 mg L⁻¹). Kinetex XB-C18 column (2.6 μm, 100 Å, 100 × 3 mm). Mobile Phase A: MeOH with 0.1% formic acid, B: 0.1% formic acid in water, pH: 3.35, flow rate: 0.25 mL min^-1^, injection volume: 1 µL, detection wavelength: 228 nm, temperature: 30 °C.

| **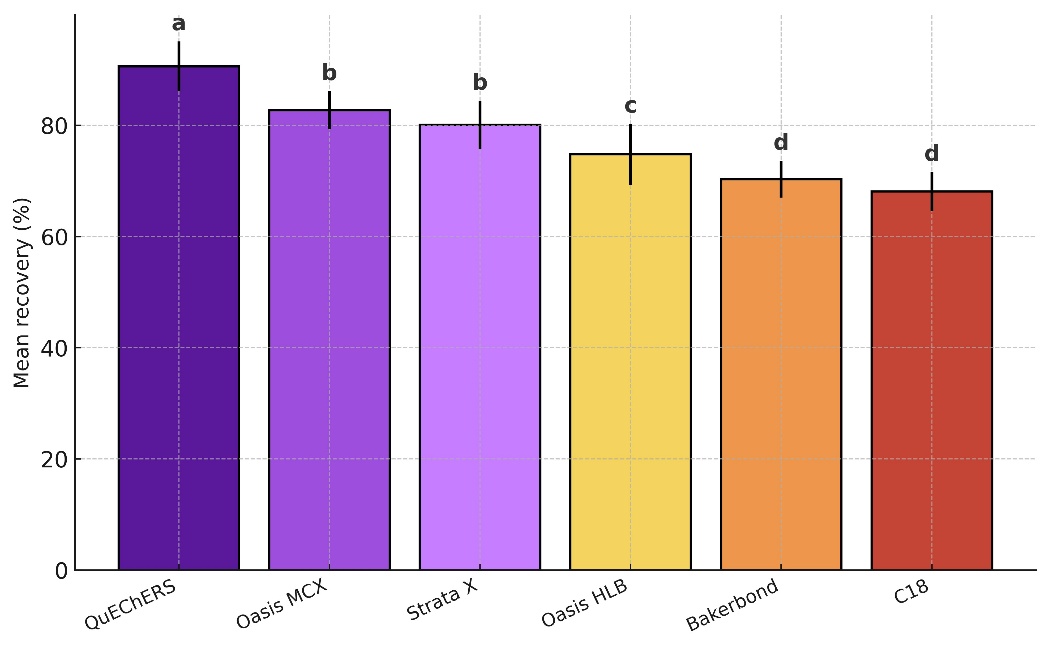** | \| **Sorbent** \| **Mean recovery (%) ± SD** \| **Group** \| \| --- \| --- \| --- \| \| **QuEChERS** \| **90.6 ± 4.5** \| **a** \| \| **Oasis MCX** \| **82.7 ± 3.4** \| **ab** \| \| **Strata X** \| **80.1 ± 4.3** \| **b** \| \| **Oasis HLB** \| **74.8 ± 5.5** \| **bc** \| \| **Bakerbond** \| **70.3 ± 3.3** \| **c** \| \| **C18** \| **68.1 ± 3.5** \| **c** \| |
| --- | --- | --- | --- | --- | --- | --- | --- | --- | --- | --- | --- | --- | --- | --- | --- | --- | --- | --- | --- | --- | --- | --- |
|  | |

| Test | F-statistic | p-value | Interpretation |
| --- | --- | --- | --- |
| One-way ANOVA | 17.38 | < 0.001 (df 5) | Significant difference among sorbents |

**Figure S6**. Tukey HSD Post-hoc Grouping (α = 0.05). Different letters within a row indicate significant differences (p < 0.05). df: degrees of freedom.

The ANOVA and Tukey grouping revealed clear differences among sorbents, with Oasis MCX falling into an intermediate statistical group (ab) and yielding recoveries closely aligned with those of the highest-performing material. For this reason, Oasis MCX was selected as the sorbent for the 3D-printed extraction device, as it provided the recovery values most comparable to the reference performance and therefore offered a suitable basis for comparison with QuEChERS.


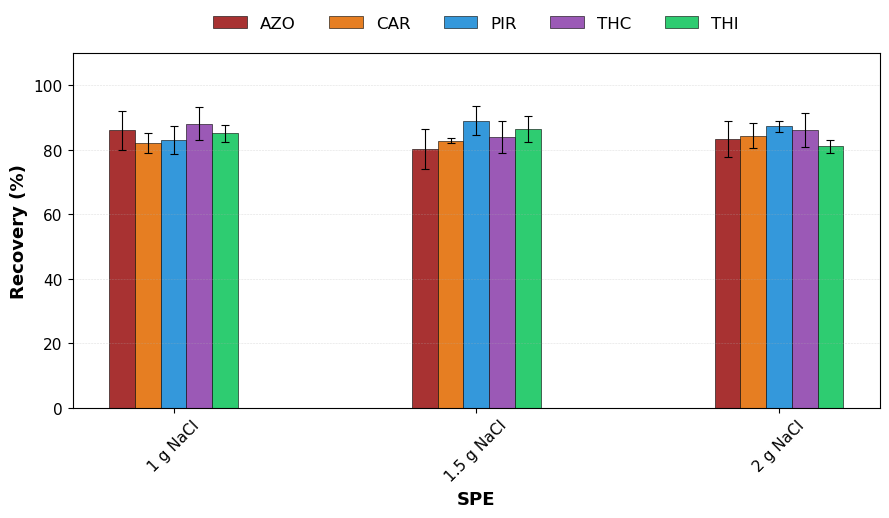


**Figure S7.** Salt addition effect on recovery. AZO: azoxystrobin, CAR: carbaryl, PIR: pirimicarb, THC: thiacloprid, THI: thiamethoxam. Results are expressed as mean recovery (%), and error bars represent standard deviation (n=3).

**Table S1.** Pesticide structure, lipophilicity value, and UV absorbance. UV absorbance (nm) values separated by commas represent wavelengths at which the compound exhibits maximum absorbance (λmax). Values separated by hyphens indicate spectral ranges where significant absorbance is observed.

| **Pesticide** | **Chemical structure** | **Lipophilicity (logP)** | **UV absorbance (nm)** |
| --- | --- | --- | --- |
| Azoxystrobin  (AZO) | 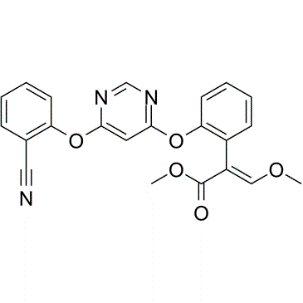 | 2.50 | 196.48 - 196.78 -197.26 |
| Pirimicarb  (PIR) | 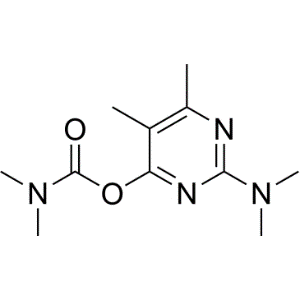 | 1.70 | 195.29, 242.41, 310.22 |
| Carbaryl  (CAR) | 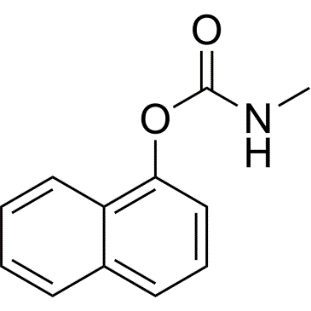 | 2.36 | 195.83, 219.53, 278.86 |
| Thiacloprid  (THC) | 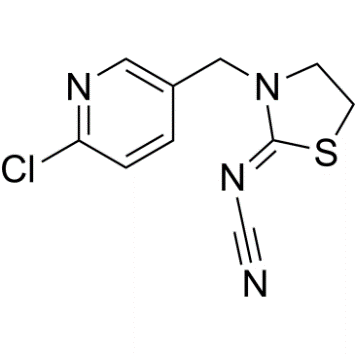 | 1.26 | 194.91, 217.71, 242.18 |
| Thiamethoxam  (THI) | 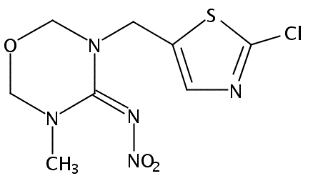 | -0.13 | 215.42 - 251.75 |

**Table S2.** Experimental design matrix of independent variables (gradient time, and temperature) with responses (resolution – Rs_min_, retention time, and plates).

| **Run** | **Gradient time (min)** | **Temperature (°C)** | **Rsₘᵢₙ** | **Retention time (min)** | **Plates** |
| --- | --- | --- | --- | --- | --- |
| 1 | 8.0 | 30.0 | 5.35 | 11.15 | 42933 |
| 2 | 8.0 | 37.5 | 6.13 | 10.19 | 51375 |
| 3 | 8.0 | 45.0 | 4.4 | 11.37 | 64688 |
| 4 | 11.5 | 30.0 | 5.99 | 9.1 | 95000 |
| 5 | 11.5 | 37.5 | 5.15 | 8.65 | 61571 |
| 6 | 11.5 | 45.0 | 5.52 | 8.65 | 62959 |
| 7 | 15.0 | 30.0 | 4.54 | 9.97 | 45438 |
| 8 | 15.0 | 37.5 | 4.69 | 8.75 | 67328 |
| 9 | 15.0 | 45.0 | 5.72 | 6.9 | 66371 |
| 10 | 8.0 | 37.5 | 4.53 | 11.21 | 60337 |
| 11 | 11.5 | 30.0 | 5.58 | 9.1 | 95000 |
| 12 | 15.0 | 37.5 | 3.79 | 6.9 | 57278 |
| 13 | 11.5 | 45.0 | 3.67 | 9.36 | 68054 |

**Table S3.** Response surface models equations for chromatographic analysis. G: gradient time (min), T: column temperature (°C).

| **Response variable** | **Model equation** |
| --- | --- |
| Minimum resolution (Rsₘᵢₙ) | Rsₘᵢₙ = 17.42 − 0.373×G − 0.492×T − 0.019×G² + 0.020×G×T + 0.003×T² |
| Average retention time (min) | Retention Time = 22.96 − 0.627×G − 0.401×T + 0.061×G² − 0.031×G×T + 0.009×T² |
| Theoretical plates | Plates = −71,013 + 36,299×G − 3,278×T − 1,539×G² − 7.83×G×T + 41.28×T² |

**Table S4.** Gradient elution time used for the chromatographic separation of five pesticides. A: mobile phase A (MeOH with 0.1% formic acid); B: mobile phase B (0.1% formic acid in water, pH 3.35).

| **Gradient time (min)** | **A (%)** | **B (%)** |
| --- | --- | --- |
| 1 | 30 | 70 |
| 7 | 70 | 30 |
| 9 | 70 | 30 |
| 11 | 30 | 70 |
| 15 | 30 | 70 |

**Table S5.** Chromatographic parameters. AZO: azoxystrobin, CAR: carbaryl, PIR: pirimicarb, THC: thiacloprid, THI: thiamethoxam.

| **Pesticide** | **Retention time (min)** | **Peak area** **(mAU-min)** | **Resolution** | **Symmetry** | **Plates** |
| --- | --- | --- | --- | --- | --- |
| THI | 3.45 | 6.066 | 28.0 | 1.14 | 7349 |
| THC | 7.58 | 3.821 | 7.6 | 1.25 | 50451 |
| PIR | 8.66 | 3.788 | 6.7 | 1.15 | 54121 |
| CAR | 9.63 | 4.046 | 16.3 | 1.14 | 72546 |
| AZO | 12.05 | 9.742 | n.a. | 1.15 | 97692 |

**Table S6.** Recovery (%) of two consecutive cycles using the same 3D-printed coated device for each replicate (n=3). AZO: azoxystrobin, CAR: carbaryl, PIR: pirimicarb, THC: thiacloprid, THI: thiamethoxam.

| Pesticide | Recovery (%)  1st cycle | Recovery (%)  2nd cycle |
| --- | --- | --- |
| AZO | 80.3 ± 3.4 | 72.4 ± 1.6 |
| CRYL | 83.7 ± 2.5 | 75.8 ± 3.2 |
| PIR | 79.0 ± 7.1 | 73.2 ± 2.5 |
| THC | 84.0 ± 2.9 | 73.6 ± 1.6 |
| THI | 86.3 ± 2.1 | 71.3 ± 1.2 |

**Table S7.** Recoveries (%) of the five pesticides in honey samples spiked at three fortification levels (31.5, 56.5, and 81.5 mg L⁻¹) using the UAE–3D-printed coated device followed by UHPLC-DAD analysis. AZO: azoxystrobin, CAR: carbaryl, PIR: pirimicarb, THC: thiacloprid, THI: thiamethoxam.

| **Pesticide** | **F-value** | **p-value** | **Interpretation** |  |
| --- | --- | --- | --- | --- |
| AZO | 4.06 | 0.077 | No significant difference |  |
| PIR | 0.38 | 0.698 | No significant difference |  |
| CAR | 0.87 | 0.465 | No significant difference |  |
| THC | 1.31 | 0.338 | No significant difference |  |
| THI | 1.10 | 0.392 | No significant difference |  |

No significant differences (p > 0.05) were observed among the three fortification levels for any of the investigated pesticides according to one-way ANOVA, confirming the consistency and robustness of the extraction performance across the tested concentration range**.**

**Table S8.** Comparison between the proposed method and the conventional QuEChERS procedure.

| **Parameter** | **Proposed method** | | **Conventional QuEChERS procedure** |
| --- | --- | --- | --- |
| Extraction principle | Ultrasound-assisted extraction using a 3D-printed SPE device coated with MCX sorbent | Liquid–liquid partitioning with MgSO₄ / NaCl salts followed by d-SPE cleanup | |
| Sample amount | 5 g of honey | 10 g of honey | |
| Solvent volume | 7.5 mL ACN + 1 mL MeOH | 10–15 mL ACN + buffer salts | |
| Salt composition | Single salt (1 g NaCl) | Mixed salts (MgSO₄ + NaCl ± citrate buffer) | |
| Extraction time | 20 min sonication + 1 min vortex | 1–5 min shaking + centrifugation | |
| Device reusability | 1–2 cycles (confirmed by SEM) | Single-use plastics and sorbents | |
| Recoveries (range) | 81–89 % | 75–95 % | |
| Solvent waste | ≈ 9 mL per sample | > 15 mL per sample | |
| Energy consumption | Low (ultrasound bath) | Moderate (centrifugation + shaking) | |
| Automation potential | Medium (manual now, adaptable to flow system) | Low (manual only) | |
| Greenness (AGREEprep score) | 0.43 – moderately green | 0.34 – lower greenness¹ | |
| Method highlights | Miniaturized, reusable, lower waste, greener, compatible with UHPLC-DAD | Established but higher waste and single-use materials | |

¹ AGREEprep value for conventional QuEChERS method (0.34) was reported in a previous study (Ravi et al., 2025).
